# Endothelial ADAM10 utilization defines a molecular pathway of vascular injury in mice with bacterial sepsis

**DOI:** 10.1172/JCI168450

**Published:** 2023-12-01

**Authors:** Danielle N. Alfano, Mark J. Miller, Juliane Bubeck Wardenburg

**Affiliations:** 1 Department of Pediatrics and; 2 Division of Infectious Diseases, Department of Internal Medicine, Washington University School of Medicine, St. Louis, Missouri, USA.

**Keywords:** Infectious disease, Vascular Biology, Bacterial infections, Endothelial cells, Thrombosis

## Abstract

The endothelium plays a critical role in the host response to infection and has been a focus of investigation in sepsis. While it is appreciated that intravascular thrombus formation, severe inflammation, and loss of endothelial integrity impair tissue oxygenation during sepsis, the precise molecular mechanisms that lead to endothelial injury remain poorly understood. We demonstrate here that endothelial ADAM10 was essential for the pathogenesis of *Staphylococcus aureus* sepsis, contributing to α-toxin–mediated (Hla-mediated) microvascular thrombus formation and lethality. As ADAM10 is essential for endothelial development and homeostasis, we examined whether other major human sepsis pathogens also rely on ADAM10-dependent pathways in pathogenesis. Mice harboring an endothelium-specific knockout of ADAM10 were protected against lethal *Pseudomonas aeruginosa* and *Streptococcus pneumoniae* sepsis, yet remained fully susceptible to group B streptococci and *Candida albicans* sepsis. These studies illustrate a previously unknown role for ADAM10 in sepsis-associated endothelial injury and suggest that understanding pathogen-specific divergent host pathways in sepsis may enable more precise targeting of disease.

## Graphical abstract

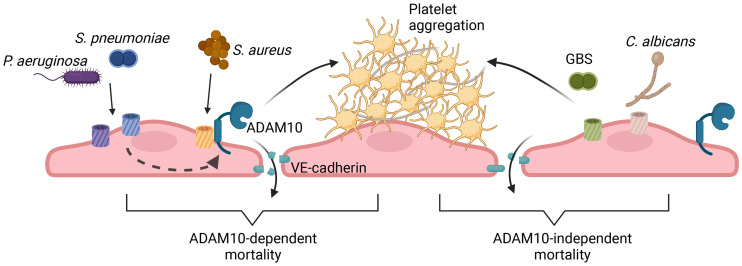

## Introduction

Bacterial sepsis is a global public health emergency, with an estimated incidence of nearly 50 million cases and 11 million deaths worldwide in 2017 (1). In the United States alone, sepsis accounts for more than $20 billion in total hospital costs (2). Sepsis is characterized by multisystem organ failure, which has been attributed to both pathogen-mediated injury and secondary insults that result from the host inflammatory response to infection (3). Despite decades of research and large-scale clinical trials of novel interventions, the mainstay of care for patients with sepsis remains limited to antibiotic administration and support of failing organs (4).

As a leading cause of human bacterial sepsis (5), *Staphylococcus aureus* invasive infections are also one of the most lethal (6). Studies of the molecular pathogenesis of *S*. *aureus* sepsis have implicated a wide array of virulence factors in evasion of the host immune response, perturbation of hemostasis, and compromise of tissue barrier integrity within the vasculature (7, 8). Among these, *Staphylococcus* *aureus* pore-forming toxins (PFTs), including α-toxin (α-hemolysin [Hla]) and an array of bicomponent leukocidins, have been the focus of intense investigation and therapeutic targeting in sepsis (9–16). Cell-type–specific injury mediated by these PFTs is dependent on the expression of distinct toxin receptors, which also define species specificity of toxin action (17). The leukocidins LukED and HlgAB are 2 widely expressed *S*. *aureus* PFTs that utilize the Duffy antigen receptor for chemokines (DARCs) as their cellular receptor (14). The leukocidin-DARC interaction contributes to vascular injury, tissue damage, and mortality, which are mitigated in an endothelial cell–specific, DARC-knockout mouse model (14). While strain-specific expression of the staphylococcal leukocidins is variable (17), Hla is expressed by nearly all clinical disease isolates and is required for the pathogenesis of *S*. *aureus* disease (10, 12, 18–20). The interaction of Hla with its cellular receptor A disintegrin and metalloprotease 10 (ADAM10) causes direct cellular injury through pore formation (18), but also leads to ADAM10 metalloprotease activation, resulting in pathologic cleavage of native ADAM10 substrates including VE-cadherin on endothelial cells (10). In the context of sepsis, Hla decreases platelet–endothelial cell adhesion through ADAM10-mediated cleavage of platelet glycoprotein VI (GPVI), compromising hemostasis but simultaneously promoting neutrophil inflammatory signaling by augmenting the platelet-neutrophil interaction (12). Both *S*. *aureus* infection and i.v. delivery of purified Hla induce platelet aggregation in the microvasculature, resulting in severe tissue injury (21). While *S*. *aureus* infection of mice harboring platelet-specific deletion of ADAM10 through PF4-mediated Cre recombinase expression (*PF4 ADAM10^–/–^*) is associated with a reduction in platelet aggregation within the liver (21) and improved intravascular hemostasis (12), these mice succumb to lethal sepsis unless ADAM10 is concurrently deleted in the myeloid cell lineage through expression of Cre recombinase under the control of the Lyz2 promoter (12).

Neutrophils and platelets cooperate to promote coagulation in the microvasculature to limit the action of invading pathogens in a process known as immunothrombosis (22). Recent studies in humans and animal models illustrate that perturbation of this immunothrombotic response can influence the outcome of the host-pathogen interaction in *S*. *aureus* sepsis. Platelets play an essential role in host defense against *S*. *aureus* infection through the production of microbicidal proteins (23). In humans, thrombocytopenia and elevated serum IL-10 levels are associated with increased severity of *S*. *aureus* sepsis and mortality, while an enhanced IL-1β response favors clearance of the pathogen from the bloodstream (24–28). Further illustrating the importance of platelet-mediated immune function, delivery of the platelet inhibitor ticagrelor improves the outcome of mice infected with *S*. *aureus* (28), and retrospective clinical data analysis suggests that ticagrelor mitigates infection risk in humans, decreasing the incidence of *S*. *aureus* bacteremia in patients receiving long-term antiplatelet therapy (29, 30). Although these data shed light on the role of platelets and inflammation in the host response to *S*. *aureus* infection, they fail to inform an understanding of the cellular mechanisms by which infection couples endothelial injury and platelet activation within the vasculature to result in immunothrombosis.

Endothelial injury is the primary physiologic stimulus for platelet activation and aggregation (31). Our findings on the role of the Hla-ADAM10 interaction in endothelial injury (10, 12), together with the observation that a SNP in the ADAM10 promoter influences the outcome of human bacterial sepsis (32), led us to hypothesize that endothelial injury by Hla may initiate the series of events that culminate in *S*. *aureus* sepsis–associated microvascular thrombosis. ADAM10 is essential in vascular cell development, homeostasis, and regeneration (33–39). While germline deletion of ADAM10 results in embryonic lethality at E9.5 (40), mice harboring endothelium-specific deletion of ADAM10 exhibit a severe abnormality of vascular development due in large part to defective Notch signaling (41). Deletion of ADAM10 by E7.5 results in lethality, however deletion after E12.5 preserves viability, but mice display abnormal blood vessels in multiple organs (42). To circumvent the impact of ADAM10 deletion on vascular development, we utilized an endothelium-specific inducible knockout system to assess the role of the metalloprotease in sepsis. Here, we demonstrate that deletion of endothelial ADAM10 provided protection against lethal *S*. *aureus* sepsis by limiting platelet aggregation and microvascular occlusion. Moreover, mice harboring endothelial ADAM10 deletion were protected against *Pseudomonas aeruginosa* and *Streptococcus pneumoniae* sepsis, yet maintained susceptibility to lethal infection with other human sepsis pathogens. These findings reveal a role for ADAM10 in molecular specificity of the host-pathogen interaction and suggest that ADAM10 may represent a promising target for sepsis therapies in a specific and definable subset of bacterial infections.

## Results

**Figure 1:**
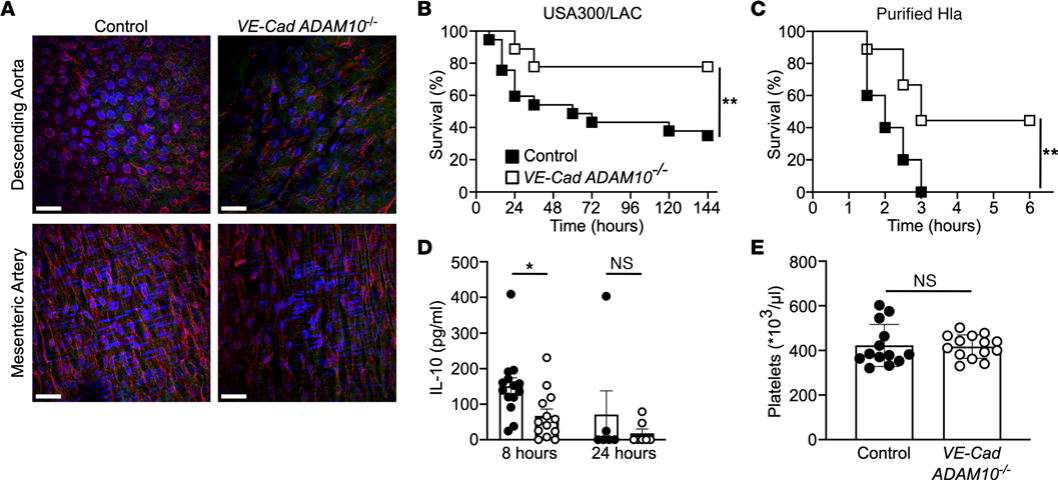
*Staphylococcus aureus* targets endothelial ADAM10 to cause lethal sepsis. (**A**) ADAM10 (red) staining in the descending aorta and mesenteric artery from control or *VE-Cad ADAM10^–/–^* mice. DAPI (blue) denotes cell nuclei; collagen autofluorescence (green). Scale bars: 30 μm. (**B**) Survival following lethal *S*. *aureus* infection in *VE-Cad ADAM10^–/–^* (*n* = 37) or control (*n* = 18) female mice. Independent experiments were repeated 4 times, and data were pooled. (**C**) Survival following lethal purified Hla sepsis in *VE-Cad ADAM10^–/–^* (*n* = 9) or control (*n* = 10) female mice. Independent experiments were repeated twice, and data were pooled. (**D**) Serum IL-10 analysis 8 and 24 hours after infection in female *VE-Cad ADAM10^–/–^* mice or controls. Data represent 3 independent pooled experiments. Data are presented as the mean ± SEM. (**E**) Mouse platelet count enumerated 4 hours after lethal infection with *S*. *aureus* in male and female mice. Data are from 3 independent pooled experiments and are presented as the mean ± SD. *P ≤ 0.05 and **P ≤ 0.01, by unpaired, 2-tailed *t* test.

To study the role of endothelial ADAM10 in *S*. *aureus* sepsis, we generated transgenic mice that enable endothelial cell–specific deletion of ADAM10 under control of the VE-cadherin promoter that drives expression of a tamoxifen-inducible (TAM-inducible) Cre recombinase (Supplemental Figure 1A; supplemental material available online with this article; https://doi.org/10.1172/JCI168450DS1) (43). ADAM10 staining of isolated aorta and mesenteric artery after TAM treatment of *VE-Cad ADAM10^loxP/loxP^* mice demonstrated a 53% and 37% reduction in *Adam10* expression, respectively, compared with that observed in vessels from control *VE-Cad ADAM10^WT/WT^* mice (Figure 1A and Supplemental Figure 1B). Although WT control mice (*VE-Cad ADAM10^+/+^*) succumbed to sepsis induced by i.v. inoculation of 1 × 10^8^ CFU *S*. *aureus* USA300/LAC, endothelial ADAM10-knockout mice (*VE-Cad ADAM10^–/–^*) were protected against lethal infection (Figure 1B). Similarly, *VE-Cad ADAM10^–/–^* mice were protected against lethality induced by i.v. delivery of purified active Hla, confirming the sufficiency of this toxin for ADAM10-dependent outcomes (Figure 1C). Evaluation of health scores (Supplemental Table 1 and Supplemental Figure 1C) and physical activity (Supplemental Videos 1 and 2) over the course of *S*. *aureus* infection reflected protection against the clinical disease observed in *VE-Cad ADAM10^–/–^* mice relative to controls, despite similar weight loss in both groups (Supplemental Figure 1D). *S*. *aureus* recovery from multiple tissues was comparable in control and *VE-Cad ADAM10^–/–^* mice 24 hours after infection (Supplemental Figure 1E), suggesting that the improved survival of *VE-Cad ADAM10^–/–^* mice was not simply related to early bacterial control.

As human mortality from *S*. *aureus* sepsis correlates with host immunologic markers of inflammation and a sustained decrease in circulating platelet numbers (24–26, 44), we evaluated serum cytokine levels and quantified platelets in WT and *VE-Cad ADAM10^–/–^* mice that received a sublethal i.v. inoculum of *S*. *aureus*. Serum IL-10 was increased in WT mice 8 hours after infection, whereas *VE-Cad ADAM10^–/–^* mice had a minimal increase in IL-10 levels (Figure 1D), mirroring the response seen in humans who survive infection. Although serum IL-1β levels were elevated 8 hours after infection, we observed no significant difference in this response between *VE-Cad ADAM10^–/–^* and control mice (Supplemental Figure 1F). Similarly, both groups of mice exhibited comparable platelet counts 4 hours after infection (Figure 1E).

**Figure 2:**
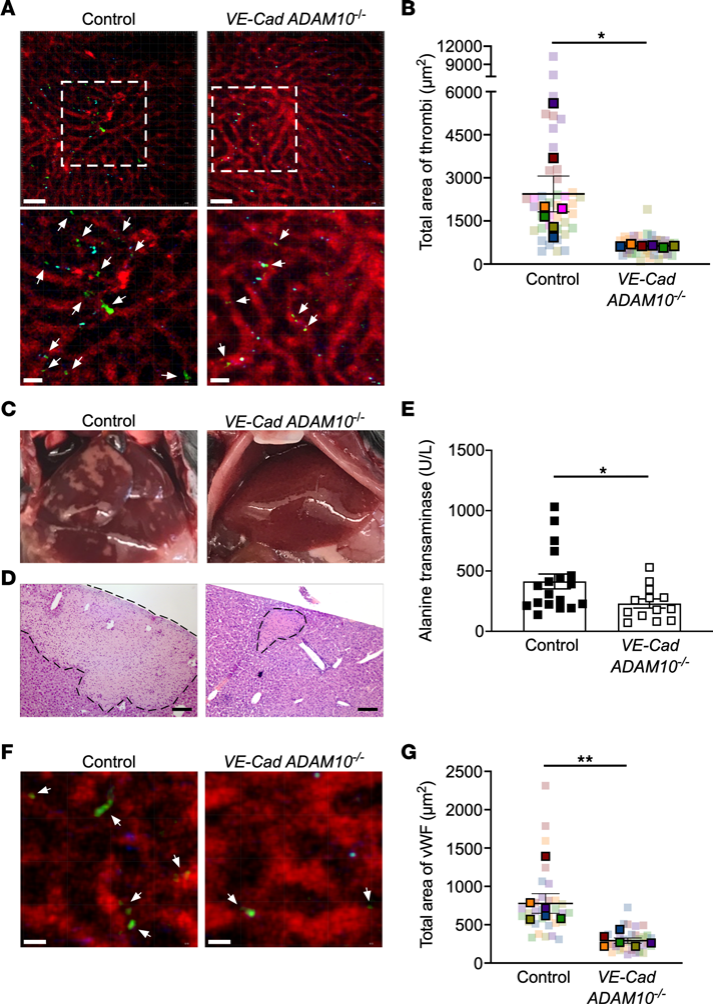
ADAM10 alters endothelial cell–platelet interactions in response to *S*. *aureus* and contributes to sepsis-associated injury. (**A**) Representative 2-photon image of control or *VE-Cad ADAM10^–/–^* mouse livers 6 to 8 hours after nonlethal *S*. *aureus* sepsis. Vasculature (red, Qdots655), platelets (green, GPIbβ). Scale bars: 50 μm. Lower panels display images outlined by a dashed line box. Scale bars: 20 μm. White arrows denote thrombi. (**B**) Quantification of the total area of platelet accumulation within the vasculature in mouse liver as treated in **A**. Data represent 5–7 FOV per mouse in control (*n* = 7F) and *VE-Cad ADAM10^–/–^* (*n* = 6F) mice. Data are presented as the mean ± SEM. Data for individual FOV within each mouse are shown in Supplemental Figure 2A. (**C**) Representative images of the liver 24 hours after *S*. *aureus* infection in control or *VE-Cad ADAM10^–/–^* mice. (**D**) H&E-stained liver sections from control or *VE-Cad ADAM10^–/–^* mice 24 hours after infection, with the area of necrosis outlined. Scale bars: 100 μm. Images in **C** and **D** are representative of 5 mice per condition from 2 independent experiments. (**E**) Serum ALT in infected control (*n* = 11 males, 7 females) or *VE-Cad ADAM10^–/–^* (*n* = 7 males, 6 females) mice 24 hours after nonlethal *S*. *aureus* infection. Data represent 3 independent pooled experiments and are presented as the mean ± SEM. (**F**) Representative 2-photon images of control or *VE-Cad ADAM10^–/–^* mouse livers 2–4 hours after lethal *S*. *aureus* sepsis. Vasculature (red, Qdots655); vWF (green). Scale bars: 10 μm. White arrows denote vWF deposition. (**G**) Quantification of the total area of vWF accumulation within the vasculature in mouse liver as treated in **F**. Data represent 5–6 FOV per mouse in control (*n* = 6F) and *VE-Cad ADAM10^–/–^* (*n* = 4 females, 2 males) mice per group and represent the mean **±** SEM. Data for individual FOV within each mouse are shown in Supplemental Figure 3F. *P ≤ 0.05 and **P ≤ 0.01, by nested *t* test for in vivo imaging (**B** and **G**) or unpaired, 2-tailed *t* test (**E**).

On the basis of these findings, we hypothesized that Hla-induced endothelial damage may incite immunothrombosis during *S*. *aureus* sepsis, coupling a functional alteration of endothelial integrity and platelet function. To inform an understanding of microvascular injury induced by *S*. *aureus* in situ, we used 2-photon microscopy to visualize platelet-endothelial interactions within the hepatic vasculature following infection of *VE-Cad ADAM10^–/–^* mice and matched controls with a nonlethal inoculum of *S*. *aureus*. WT mice developed more substantial platelet aggregates than did *VE-Cad ADAM10^–/–^* mice 6–8 hours after infection (Figure 2, A and B, and Supplemental Figure 2A). We observed a limited thrombus burden in both control and *VE-Cad ADAM10^–/–^* uninfected mice (Supplemental Figure 2B), which was statistically different from that observed in infected *VE-Cad ADAM10^–/–^* mice (average thrombi area of 104 μm^2^ vs. 635 μm^2^, SEM ± 97.93, *P* < 0.0001). Mice with platelet-specific ADAM10 deletion (*PF4 ADAM10^–/–^*) were also protected from platelet aggregate formation within the liver vasculature, whereas mice with myeloid-specific ADAM10 deletion (*LysM ADAM10^–/–^*) were not protected (Supplemental Figure 2, C and D). As neither *PF4 ADAM10^–/–^* nor *LysM ADAM10^–/–^* mice are protected from lethal *S*. *aureus* sepsis (12), these data together suggest that microvascular injury and platelet aggregation are intimately linked to impair tissue oxygenation and organ function (45, 46). We thus examined end-organ injury in *VE-Cad ADAM10^–/–^* mice. Although gross pathologic analysis of control livers revealed large areas of ischemia and necrosis (Figure 2C), *VE-Cad ADAM10^–/–^* mice had a markedly lesser degree of injury following infection that was also evident on histopathologic analysis of the liver (Figure 2D) and in quantification of serum alanine aminotransferase levels (Figure 2E). We did not detect differences in liver IL-1β or IL-10 levels after infection (Supplemental Figure 2, E and F), suggesting that local inflammation alone is not readily equated with the end-organ tissue damage observed.

Our hypothesis that endothelial injury caused by Hla initiates a pathologic platelet response within the vasculature was consistent with findings in a recent study by Sun et al. demonstrating that treatment with ticagrelor, a platelet P2Y12 inhibitor, protected against *S*. *aureus* sepsis (28). Ticagrelor blocked Hla-mediated platelet cytotoxicity in an ADAM10-dependent manner in vitro, allowing for improved clearance of the pathogen. Consistent with these findings, WT mice treated with ticagrelor in our model system exhibited a significant reduction in platelet aggregation within the hepatic vasculature compared with vehicle-treated controls (Supplemental Figure 3, A and B). As ticagrelor has been shown to exhibit pleiotropic effects beyond platelet inhibition (47), we examined whether ticagrelor affected endothelial ADAM10 activation and endothelial dysfunction. Hla-induced microvascular injury leads to barrier disruption and vascular permeability (10), inducing the deposition of von Willebrand factor (vWF) on the endothelium (12). vWF is critical in hemostasis, mediating initial platelet tethering under fluid shear stress in the vasculature (48). Treatment of human pulmonary artery endothelial cells (HPAECs) with purified Hla led to increased ADAM10 activity (Supplemental Figure 3C) and LDH release indicative of cellular injury (Supplemental Figure 3D), which was abrogated by pretreatment of HPAECs with the specific ADAM10 inhibitor GI254023X. Pretreatment of HPAECs with ticagrelor did not modulate ADAM10 activation (Supplemental Figure 3C) or LDH release induced by Hla (Supplemental Figure 3D); similarly, ticagrelor did not prevent VE-cadherin cleavage or vWF extrusion in response to Hla (Supplemental Figure 3E), but these were ablated by pretreatment with GI254023X. Thus, we conclude that the effects of ticagrelor on *S*. *aureus* sepsis appeared to be focused on the modulation of platelet function, which we hypothesize is downstream of endothelial injury elicited by the pathogen.

To assess the temporal nature of endothelial injury and platelet aggregation in vivo, we used 2-photon microscopy to visualize vWF deposition within the hepatic vasculature during *S*. *aureus* sepsis in *VE-Cad ADAM10^–/–^* mice or matched controls. Within 4 hours of infection, WT mice developed increased vWF deposition compared with *VE-Cad ADAM10^–/–^* mice (Figure 2, F and G, and Supplemental Figure 3F) preceding the difference in platelet aggregation seen at 6–8 hours (Figure 2, A and B). Together, these findings suggest a sequence of events in the microvasculature in which Hla-mediated endothelial injury functioned as an inciting event, leading to the presentation of vWF on the injured endothelium as a platelet tethering site. This pathogenic sequence presents 2 distinct therapeutic opportunities to combat sepsis: protecting the endothelium from initial injury and preventing platelet aggregation that is catalyzed by this injury.

**Figure 3:**
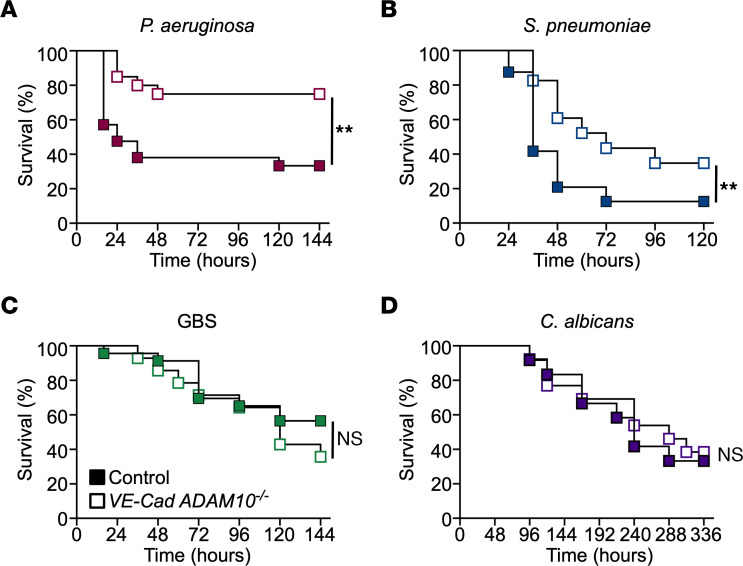
Endothelial ADAM10 mediates lethal sepsis in a pathogen-specific manner. Survival curves for control and *VE-Cad ADAM10^–/–^* mice infected with lethal *P*. *aeruginosa* (**A**, *n* = 21 [13 males, 8 females], *n* = 20 [11 males, 8 females]); *S*. *pneumoniae* (**B**, *n* = 24 [8 males, 16 females], *n* = 23 [11 males, 12 females]; GBS (**C**, *n* = 23 [9 males, 14 females], *n* = 14 [7 males, 7 females]; or *C*. *albicans* (**D**, *n* = 12 males, 13 males ). **P ≤ 0.01, by log-rank (Mantel-Cox) test for survival curves.

**Figure 4:**
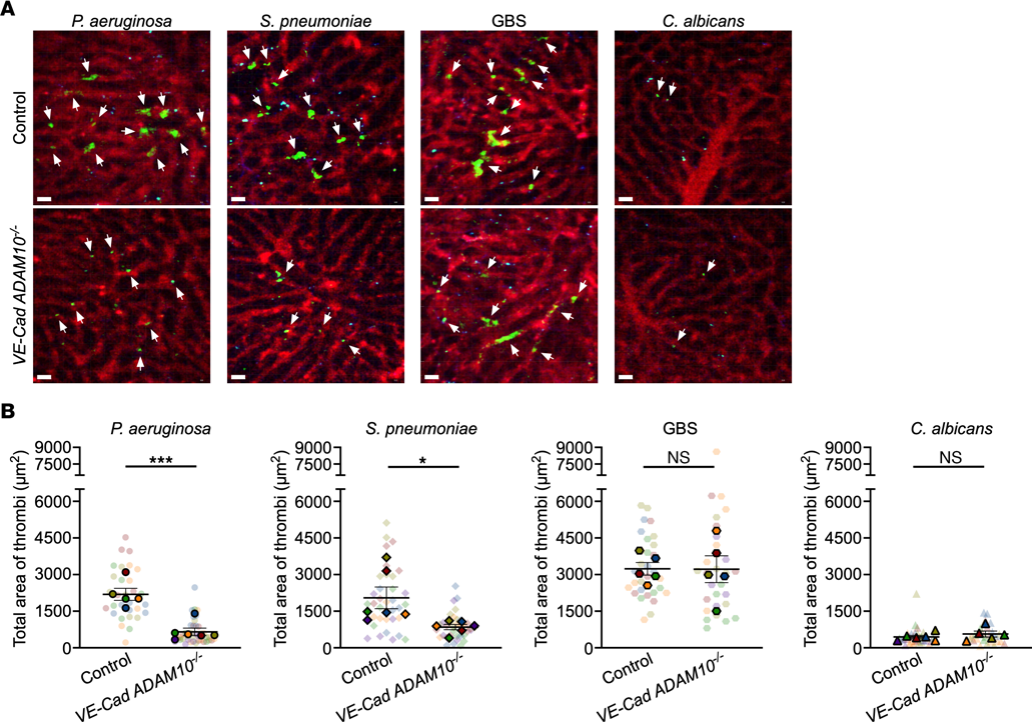
Endothelial ADAM10 mediates platelet aggregation in a pathogen-specific manner. (**A**) Representative 2-photon images of control and *VE-Cad ADAM10^–/–^* mouse livers at 6–8 hours and (**B**) corresponding quantification of the total area of platelet accumulation within the vasculature of mouse livers after *P*. *aeruginosa*, *S*. *pneumoniae*, GBS, or *C*. *albicans* sepsis. Vasculature (red, Qdots655); platelets (green, GPIbβ). Scale bars: 20 μm. White arrows denote thrombi. Data represent 5–7 FOV per mouse in 5–6 male and female mice per group and indicate the mean ± SEM. Measurements for individual FOV within each mouse are displayed in Supplemental Figure 4, E–H. *P ≤ 0.05 and ***P ≤ 0.001, by nested *t* test.

**Figure 5:**
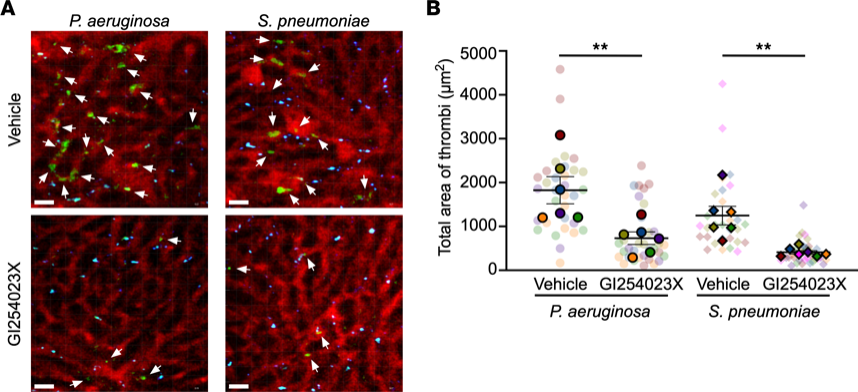
ADAM10 mediates platelet aggregation. (**A**) Representative 2-photon images of C57Bl/6 mice 6–8 hours after *P*. *aeruginosa* or *S*. *pneumoniae* sepsis, pretreated for 3 days with vehicle or the ADAM10 inhibitor GI254023X. Vasculature (red, Qdots655), platelets (green, GPIbβ). Scale bar: 20 μm. White arrows denote thrombi. (**B**) Quantification of the total area of platelet accumulation within the vasculature in mouse livers as treated in **A**. Data represent 5–7 FOV per mouse in 6–7 male and female mice per group and are presented as the mean ± SEM. Measurements for individual FOV within each mouse are displayed in Supplemental Figure 5, A and B. **P ≤ 0.01, by nested *t* test for each treatment group.

Endothelial injury is not unique to *S*. *aureus* disease, as it has been studied in the molecular pathogenesis of experimental sepsis in multiple models. The clinical manifestations of endovascular injury and progression of severe sepsis exhibit an unexpected homogeneity across distinct infectious pathogens, suggesting that pathogen-specific pathways that incite injury trigger a convergent biologic process in the host. We considered whether ADAM10 may be an upstream molecular pathway in sepsis pathogenesis, especially given the observation that an ADAM10 promoter polymorphism modulates sepsis severity (32). Three other bacterial cytotoxins have been linked to ADAM10 activation in vitro. We previously demonstrated that treatment of alveolar epithelial cells with *S. pneumoniae* pneumolysin (PLY), a cholesterol-dependent cytolysin, elicited ADAM10-mediated cleavage of epithelial cadherin (E-cadherin) (19). PLY is a PFT produced by nearly all clinical isolates and is required for virulence in invasive infection (49). Formation of the PLY pore on endothelial cells causes calcium influx, thereby activating phospholipase A2 (50) and stimulating vWF secretion (51). Reboud et al. demonstrated that *Pseudomonas aeruginosa* PFT exolysin (ExlA) and *Serratia marcescens* hemolysin (ShlA) trigger ADAM10 activation by promoting Ca^2+^ influx into the endothelial cell, leading to VE-cadherin cleavage (52). *P*. *aeruginosa* also disrupts endothelial barrier integrity through the secreted elastase LasB (53), whereas the type 3 secretion system (T3SS) effector ExoU exhibits phospholipase A2 activity, rapidly inducing membrane injury (54, 55) and triggering vWF release from endothelial cells in vitro (56). Among *P*. *aeruginosa* strains, expression of ExlA or the T3SS appear to be mutually exclusive mechanisms by which endothelial cells are injured (55, 57, 58). The ability of diverse bacterial cytotoxins to elicit endothelial injury, together with the central role of ADAM10 in endothelial homeostasis, led us to hypothesize that ADAM10 is a central mediator of sepsis-induced endothelial dysfunction. To address this, we initially focused on *P*. *aeruginosa*, *S*. *pneumoniae*, group B *Streptococcus* (GBS), and *Candida albicans*, which are leading causes of sepsis in pediatric and adult populations (59, 60). Like *S*. *aureus*, *P*. *aeruginosa*, and *S*. *pneumoniae*, both GBS and *C. albicans* harbor cytotoxins that cause membrane injury. GBS β-hemolysin/cytolysin is a cytolytic pigment that plays a key role in GBS pathogenesis, resulting in membrane injury in multiple cell types that govern barrier stability and the host immune response (61). Candidalysin, the PFT of *C*. *albicans*, compromises barrier integrity by uncontrolled Ca^2+^ influx leading to a proinflammatory cascade (62, 63). Upon infection of WT and *VE-Cad ADAM10^–/–^* mice with these pathogens, *VE-Cad ADAM10^–/–^* mice were protected against lethal sepsis caused by *P*. *aeruginosa* (Figure 3A) and *S*. *pneumoniae* (Figure 3B), but not sepsis due to GBS (Figure 3C) or *C*. *albicans* (Figure 3D). Clinical health scores during *P*. *aeruginosa* and *S*. *pneumoniae* infection mimicked those observed in *VE-Cad ADAM10^–/–^* mice during *S*. *aureus* sepsis (Supplemental Figure 4, A–D). We next assessed whether platelet aggregation within the hepatic vasculature was altered in an ADAM10-dependent manner following infection with these pathogens. In vivo imaging of *VE-Cad ADAM10^–/–^* mice infected with *P*. *aeruginosa* or *S*. *pneumoniae* revealed significantly reduced areas of platelet aggregation in the liver 6–8 hours after infection compared with control WT mice (Figure 4, A and B, and Supplemental Figure 4, E and F). In contrast, platelet thrombus formation was indistinguishable in control and *VE-Cad ADAM10^–/–^* mice after infection with GBS or *C*. *albicans* (Figure 4, A and B, and Supplemental Figure 4, G and H). To further investigate the role of endothelial ADAM10 as an inciting factor in microvascular injury in vivo following infection with *P*. *aeruginosa* or *S*. *pneumoniae*, we examined whether the use of the ADAM10 active site inhibitor GI254023X could prevent pathogen-induced platelet aggregation on the injured endothelium. C57Bl/6 mice were pretreated for a 3-day period with GI254023X via i.p. injection and then infected with either *P*. *aeruginosa* or *S*. *pneumoniae* and imaged as described above. We found that treatment with the ADAM10 inhibitor protected against platelet aggregation within the hepatic vasculature in both *P*. *aeruginosa* and *S*. *pneumoniae* sepsis in a manner similar to that observed in *VE-Cad ADAM10^–/–^* mice (Figure 5, A and B, and Supplemental Figure 5, A and B). Together, these studies provide the first in vivo evidence to our knowledge that the role of endothelial ADAM10 in the pathophysiology of sepsis extends beyond that of the known Hla-ADAM10 complex, exhibiting pathogen specificity.

**Figure 6:**
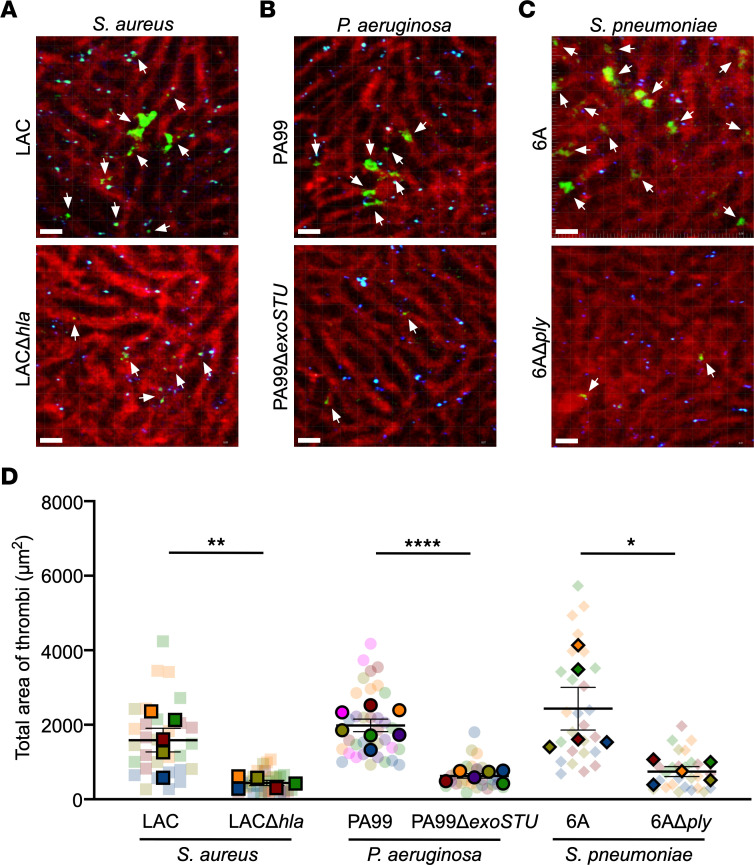
Bacterial cytotoxins mediate sepsis-induced platelet aggregation in the liver. Representative 2-photon images of C57Bl/6 mice 6–8 hours after sepsis with (**A**) *S*. *aureus* LAC or LACΔ*hla*, (**B**) *P*. *aeruginosa* PA99 or PA99Δ*exoSTU*, and (**C**) *S*. *pneumoniae* 6A or 6AΔ*ply*. Vasculature (red, Qdots655); platelets (green, GPIbβ). Scale bars: 20 μm. White arrows denote thrombi. (**D**) Quantification of the total area of platelet accumulation within the vasculature in mouse livers as treated in **A**–**C**. Data represent 5–7 FOV per mouse in 5–7 female mice per group and are presented as the mean ± SEM. Measurements for individual FOV within each mouse are displayed in Supplemental Figure 5, C–E. *P ≤ 0.05, **P ≤ 0.01, and ****P ≤ 0.0001, by nested *t* test for each treatment group.

Like *S*. *aureus*, many pathogens utilize PFTs or other membrane-injurious cytotoxins to damage and manipulate the endothelium (54, 64–66), raising the possibility that these virulence factors may couple intravascular infection with the observed dependence on ADAM10. PFTs and other membrane-active cytotoxins generally have 2 main effects during infection: the compromising of barrier integrity and disruption of the host immunological response (64, 67, 68). On the basis of our observation that microvascular occlusion in response to endothelial injury distinguished *S*. *aureus*, *P*. *aeruginosa*, and *S*. *pneumoniae* from pathogens that exhibited ADAM10 independence, we leveraged the 2-photon in vivo imaging model of platelet aggregation to examine the impact of membrane-active cytotoxins on disease pathogenesis. Upon infection of C57Bl/6 mice with WT *S*. *aureus* LAC or an isogenic Hla-deficient mutant (LACΔ*hla*), we confirmed that platelet aggregation in response to *S*. *aureus* sepsis was dependent on Hla (Figure 6, A and D, and Supplemental Figure 5C). We then extended these studies to determine whether microvascular occlusion in *P*. *aeruginosa* and *S*. *pneumoniae* infection was dependent on cytotoxin expression. We used WT *P*. *aeruginosa* PA99 or its isogenic strain PA99Δ*exoSTU*, which is impaired in effector secretion without disruption of expression of the T3SS (69), and WT *S*. *pneumoniae* 6A or its isogenic PLY-deficient mutant (6AΔ*ply*). Compared with their WT strains, both toxin mutant strains elicited a reduction in platelet aggregation within the hepatic vasculature 6–8 hours after infection (Figure 6, B–D, and Supplemental Figure 5, D and E). Together, these data suggest that specific bacterial cytotoxins may either initiate or amplify ADAM10-dependent endothelial injury, resulting in platelet aggregation and microvascular thrombosis in the pathogenesis of sepsis.

## Discussion

Interventional trials to mitigate sepsis-associated mortality have centered around agents that stabilize the endothelium, dampen inflammation, or curtail microvascular thrombosis (70–74). The failure of each of these clinical trials suggests that a universal therapy for sepsis is incongruent with the molecular heterogeneity of the host-pathogen interaction. Our study not only sheds light on the specific role of the Hla-ADAM10 interaction in endothelial injury in *S*. *aureus* sepsis, but, perhaps more importantly, illustrates that utilization of ADAM10 differentiates molecular pathways of disease among common human sepsis pathogens.

Multiple lines of evidence now indicate that the endothelial cell is a primary target of virulence factor–mediated injury in *S*. *aureus* sepsis and suggest that a temporal sequence of events unfolds in the pathogenesis of intravascular infection. *S*. *aureus* mutants deficient in bacterial adhesion and clumping demonstrate a severe defect in intravascular infection (75, 76), implicating the formation of a bacterial nidus within the microvasculature as a nucleating event that in turn may facilitate localized action of the staphylococcal PFTs on the endothelium. As a physiologic response to endothelial injury, platelet aggregation and focal leukocyte activation may then amplify the microvascular insult, resulting in injury to the tissues supplied by the afflicted vascular beds. This model is consistent with the finding that endothelium-specific knockout of DARC or ADAM10 alone affords protection against lethal sepsis, whereas a more modest effect of ADAM10 knockout on platelets and myeloid lineage is observed, requiring protection of both cell types to prevent mortality in experimental *S*. *aureus* sepsis (12, 21).

The use of cytotoxins is a highly conserved mechanism by which pathogens elicit host tissue injury. Although diverse in structure and mechanism of action, PFTs and membrane-associated cytotoxins cause endothelial barrier damage, inflammasome activation, alterations in vascular tone, and immune cell injury by manipulation of host signaling pathways (55, 64, 67, 68). As we observe discriminatory use of ADAM10 by the pathogens we examined, it will be essential to extend these studies toward an understanding of the precise molecular mechanisms by which ADAM10 engenders cytotoxin-mediated injury. There are several plausible mechanisms by which pathogen-specific dependence on ADAM10 may occur. ADAM10 exhibits inducible catalytic activity, which leads to metalloprotease-dependent cleavage of ectodomain-containing substrates including VE-cadherin within the vasculature (77). Both ADAM10 translocation to the cell surface and catalytic activation have been observed to be calcium dependent (34, 78). As multiple PFTs induce Ca^2+^ influx, including those expressed by GBS and *C*. *albicans* (62–64, 79), the induction of Ca^2+^ signaling alone is unlikely to be the singular mechanism by which ADAM10-dependent pathogens elicit microvascular injury. It is possible, however, that ADAM10-dependent pathogens target specific membrane microdomains and engender cellular signal transduction events dependent on ADAM10, or alternatively specify the cleavage of a distinct subset of ADAM10 substrates to amplify endovascular pathogenesis. Recent structural insights have illustrated that spatial context is pivotal in the selection of ADAM10 substrates for cleavage (80). The anatomic and functional heterogeneity of the endothelium between distinct vascular beds as well as within individual organs (81, 82) may thus contribute to site-specific ADAM10-dependent injury or cleavage events. To differentiate between these mechanisms, dedicated analyses of virulence factor expression and tropism of individual pathogens for biologically distinct sites within the vasculature, along with detailed analysis of ADAM10 activation and downstream signaling events, will need to be conducted.

Several limitations of mouse modeling systems may obscure a complete understanding of the role of ADAM10 in pathogen-specific outcomes in sepsis. First, the expression level of ADAM10 on the murine endothelium may be distinct in magnitude or vascular distribution relative to that in humans. The ADAM10 rs653765 promoter polymorphism appears to regulate protein expression, suggesting a mechanism by which the SNP may influence the outcome of human bacterial sepsis (32, 83). Additional studies will be needed to reveal whether the effects of this polymorphism in vivo stem solely from endothelial ADAM10 function or also depend on other cell types in which ADAM10 may contribute to sepsis pathogenesis. While we did not observe differences in sepsis outcomes in *VE-Cad ADAM10^–/–^* mice following infection with GBS or *C*. *albicans*, subtle differences may exist that exceed the sensitivity of detection in a mouse model system. Similarly, ADAM10 expression on other cell types may contribute to disease caused by GBS or *C*. *albicans*. Additional mechanistic studies of the role of ADAM10 in sepsis and the contribution of SNP-based regulation of ADAM10 expression in disease are needed.

Moreover, our findings suggest that further investigation of cytotoxin activity across a broader array of leading sepsis pathogens from the Enterobacteriaceae, Clostridiaceae, and Streptococcaceae families may expand our understanding of ADAM10 dependence in pathogenesis. This knowledge has several important implications for clinical disease. First, these studies provide a molecular basis to indicate that therapeutic targeting of host pathways in sepsis can be defined in a pathogen-specific manner. This type of “precision medicine” approach is not currently achievable with our current understanding of sepsis pathogenesis. The independent effects of genetic alteration of ADAM10 expression and ADAM10 inhibition on sepsis mortality and vascular injury highlight the potential use of small-molecule inhibitors as a novel host-targeted approach to modify disease. The use of active-site ADAM10 inhibitors such as GI254023X has shown promising preclinical results to improve sepsis (10, 19); however, the evaluation of these agents in humans remains limited by nonspecific inhibitory effects on ADAM17 and unfavorable toxicities with chronic use (84, 85). Newer compounds with improved selectivity for ADAM10 have shown promise in preclinical cancer models, with improved patient tolerance (84). In addition, highly specific anti-ADAM10 monoclonal antibodies have been shown to block ADAM10-specific substrate cleavage in vitro and in a mouse xenograft model (86, 87). The selectivity of these newer agents, coupled with short-term treatment needed for sepsis, may improve the therapeutic potential for this class of host-targeted interventions.

Our findings provide the rationale for pathogen-specific analysis of the ADAM10 rs653765G promoter SNP in individuals presenting with sepsis. While prior studies have been limited to analysis of sepsis severity across cohorts of patients with sepsis, a refinement of clinical investigation to include ADAM10 SNP typing for pathogen-specific analyses may be highly informative. The pairing of these human studies with mechanistic analyses in tractable animal models may in turn provide a foundation for clinical risk stratification in sepsis and facilitate therapeutic targeting of ADAM10.

Second, our studies lend support for further investigation of the role of antiplatelet agents in the management of sepsis, raising the possibility that this class of drugs may benefit individuals infected with pathogens that rely on endothelial ADAM10 for induction of microvascular thrombosis. Recent retrospective and matched cohort studies in humans with bacteremia or sepsis have provided evidence of improved clinical outcomes among patients already receiving antiplatelet therapy for management of cardiovascular disease (88, 89). Our studies involving pretreatment of mice with ticagrelor, a commonly prescribed FDA-approved antithrombotic drug, showed that platelet aggregation was prevented within the hepatic vasculature during *S*. *aureus* sepsis, similar to what we observed with *VE-Cad ADAM10^–/–^* mice. Pretreatment with ticagrelor also demonstrated a survival benefit in mice with *S*. *aureus* sepsis (28), which is not seen in platelet-specific ADAM10-knockout mice (12), suggesting that the effects of ticagrelor were broadly protective against the multiple mechanisms by which *S*. *aureus* mediates platelet injury (30, 90). Despite our data showing that ticagrelor did not protect against endothelial vWF extrusion or VE-cadherin cleavage in vitro, ticagrelor treatment both in vitro and in vivo attenuated tissue factor expression, suggesting that ticagrelor may have other endothelial-specific antithrombotic properties (91). It is plausible that combination therapy achieving ADAM10 inhibition and antiplatelet activity may be particularly effective in the management of sepsis caused by those pathogens that elicit complex microvascular injury through an ADAM10-dependent mechanism.

The failures of clinical trials in sepsis over the past 30 years (70–74) have highlighted a need to reexamine patient stratification and sepsis treatments in clinical trials. Here, we define a pathogen-specific role of ADAM10 in sepsis, allowing us to begin to shift the paradigm from a single sepsis pathway toward an understanding of pathogen-defined pathways as the primary inciting event in cellular and tissue dysregulation. Understanding the precise molecular mechanisms by which specific pathogens exploit ADAM10 as a central pathway regulator is expected to support consideration of novel targeted therapeutics.

## Methods

### Animals.

*VE-Cad-Cre-ER^T2^–*transgenic C57Bl/6 mice (43) were bred with *Adam10^loxP/loxP^*-transgenic C57Bl/6 mice to generate *VE-Cad-Cre-ER^T2^ Adam10^loxP/loxP^* mice (*VE-Cad ADAM10^–/–^*). *VE-Cad-Cre-ER^T2^ Adam10^+/+^* mice were used as controls. Mice were genotyped using genomic DNA from the ear. The presence of *VE-Cad-Cre-ER^T2^* in mice was detected by PCR as previously described (43). *Adam10^loxP/loxP^* was detected by PCR as previously detailed (19). Endothelium-specific knockout was induced by i.p. injection of 4- to 5-week-old mice with 2 mg TAM (MilliporeSigma, T5648) daily over 5 consecutive days. Both WT and *VE-Cad ADAM10^–/–^* mice received TAM injections. ADAM10 myeloid lineage–knockout mice (*LysM ADAM10^–/–^*) (20) and platelet-specific knockout mice (*PF4 ADAM10^–/–^*) (12) have been previously described. Mice were housed under a 12-hour light/12-hour dark cycle with ad libitum access to food and water in a specified pathogen–free facility.

### Arterial vessel whole-mount and immunofluorescence.

Following euthanasia, the descending thoracic aorta and mesenteric arteries were excised from 7-week-old *VE-Cad ADAM10^–/–^* and age-matched control mice that had been flushed with PBS, cut open longitudinally, and placed in PBS. Vessels were then incubated in blocking buffer (PBS with 3% BSA, 1% fish gelatin, 0.5% Triton X-100) for 1 hour followed by a primary anti–mouse Adam10 ectodomain antibody (5 μg/mL, R&D Systems, AB946) at 4°C overnight and then a secondary antibody, Alexa Fluor 594 donkey anti-goat (1:500, Life Technologies, Thermo Fisher Scientific, A11058) for 1 hour at room temperature. DAPI (1:2,000, Thermo Fisher Scientific, 62248) was used for nuclear labeling. Vessels were mounted on a glass slide with ProLong Gold Antifade Reagent (Thermo Fisher Scientific) and cured overnight. *Z*-stack images of the endothelial cell layer were obtained with a FV1000 Olympus Confocal microscope and FV10-ASW 3.0 software at ×60 magnification. Using ImageJ-Fiji (NIH) and a LungJ plugin (92), a maximum intensity image was created from the *Z*-stack, and fluorescence was measured by mean red pixel intensity. Statistical analysis was done taking the average of different regions of the arteries per mouse with 3 mice per group.

### Microbial strains and culturing.

The *S. aureus* strains USA300 LAC and LAC *hla:erm* (LACΔ*hla*) (93) were inoculated directly from frozen stock into tryptic soy broth (TSB) medium and grown overnight at 37°C while shaking. The *P. aeruginosa* strains PA99 and PA99Δ*exoSTU* (69) were streaked from frozen cultures onto Luria-Bertani (LB) agar. A single colony was grown in MINS medium (25 mM KH_2_PO_4_, 95 mM NH_4_Cl, 50 mM monosodium glutamate, 110 μM disodium succinate, 10 mM trisodium nitrilotriacetic acid, 2.5% glycerol, 5 mM MgSO_4_, 18 μM FeSO_4_) at 37°C overnight with shaking. The *S. pneumoniae* strains P1547, serotype 6A (WT strain), and 2436 6AΔ*ply* strain (94) were streaked from frozen cultures onto a tryptic soy agar (TSA) blood agar plate. The GBS strain COH-1 was streaked from frozen cultures onto a TSA plate. A single colony was grown in Todd Hewitt broth (THB) medium at 37°C, standing overnight. The *C. albicans* strain SN425 was streaked from frozen cultures onto yeast extract peptone (YEP) agar. A single colony was grown overnight in YEP medium at 30°C with shaking.

### Sepsis modeling.

Animal experiments were carried out using 7- to 8-week-old age- and weight-matched female and male mice. For survival studies, a murine sepsis score system was used to monitor the mice on the basis of their appearance, level of consciousness, activity, and response to stimuli (Supplemental Table 1). Mice were anesthetized prior to infection with ketamine and xylazine or isoflurane inhaled gas.

For infection experiments, *S*. *aureus* LAC was subcultured without antibiotics, and *S*. *aureus* LACΔ*hla* was subcultured in the presence of 100 μg/mL erythromycin until the exponential phase (OD_600_, 0.5), washed once with PBS, and resuspended in PBS to deliver an inoculum of 0.8 to 1 × 10^8^ bacteria/100 μL via retro-orbital i.v. injection for lethal infections and 5 × 10^7^ bacteria/100 μL for sublethal infections. Purified recombinant Hla was resuspended in PBS to deliver an inoculum of 2.5–3 μg per mouse via retro-orbital i.v. injection for lethal infection. *P*. *aeruginosa* was subcultured in fresh MINS medium, regrown to the exponential phase, and resuspended in PBS to deliver an inoculum of approximately 3 × 10^7^ bacteria/100 μL via i.p. injection. *S*. *pneumoniae* entire lawn from TSA blood agar plate was grown in TSB at 37°C with 5% CO_2_ until the exponential phase (OD_620_, 1.0), washed once with PBS, and resuspended in PBS to deliver an inoculum of 2 × 10^2^ to 4 × 10^2^ bacteria/100 μL via retro-orbital i.v. injection. GBS was subcultured into fresh THB medium, regrown to the exponential phase (OD_600_, 0.4), and resuspended in PBS to deliver an inoculum of 1 × 10^8^ to 3 × 10^8^ bacteria/100 μL via retro-orbital i.v. injection. *C*. *albicans* was subcultured into fresh YEP medium and regrown for 4 hours to the log phase. Cells were washed twice in PBS and then counted with a hemocytometer to determine cell numbers for the inoculum. The culture was resuspended to deliver an inoculum of 1 × 10^5^ cells/100 μL via i.v. injection.

### Tissue and blood analysis.

Lungs perfused via the right ventricle with 4 mL PBS, or liver, heart, kidney, and spleen tissues were homogenized in 1 mL PBS with 1.0 glass/silica bead mix using bead beater homogenizer for CFU enumeration by serial dilution plating or by ELISA for cytokine analysis. Blood CFU were analyzed from whole blood following cardiac puncture by serial dilution plating. For histopathologic studies, H&E staining was performed on 10% neutral buffered formalin–fixed tissues. Blood was collected in a heparinized syringe (or an acid citrate dextrose–coated syringe for platelet counts) by cardiac puncture. Samples were centrifuged for the retrieval of plasma. Alanine transaminase (ALT) levels in plasma and the platelet count in whole blood were analyzed by Washington University in St. Louis Division of Comparative Medicine Research Animal Diagnostic Laboratory. IL-10 and IL-1β levels were analyzed using commercial ELISA kits according to the manufacturer’s protocols (R&D Systems).

### In vivo imaging.

Mice were infected as described above. At designated time points after infection, in vivo 2-photon imaging of liver was performed using a video-rate 2-photon microscope with a custom imaging chamber as previously described (95). Briefly, mice were anesthetized with isoflurane, and the liver was surgically exposed with an incision running along the base of the rib cage. A single liver lobe was carefully attached to a coverslip using VetBond (3M). A 7 μL mixture of DyLight 488 anti–mouse GPIbβ (70 μL, Emfret Analytics, X488) or FITC polyconal anti-vWF (Emfret Analytics, P150-1) and Q-tracker 655 (Thermo Fisher Scientific, Q2102MP) was injected retro-orbitally to label platelet aggregates or vWF deposits and blood vessels, respectively. The mouse was positioned in the imaging chamber so that the liver lobe was visible through the cover glass in the upper plate. The upper plate was lowered until contact was made with the liver. Both upper and lower plates were warmed using a Warner Dual Channel temperature controller to maintain core body temperature. Perfusion of hepatic vessels was confirmed by reflected light microscopy and then monitored by observing Q-tracker flow during 2-photon imaging. Fluorescence was excited with a Chameleon Vision II Ti:Sapphire laser (Coherent) tuned to 900 nm and 480 nm; 560 nm and 635 nm emission filters were used to detect collagen in blue (<480 nm), platelets or vWF in green (480–560 nm), and blood vessels in red (>635 nm). To document vessel flow and aggregation dynamics, we streamed 250 full-frame images (512 × 512, 0.8 pixels/μm) at 10 frames/s with a 3-frame average. For each animal, platelet aggregates or vWF deposits were analyzed in 5–7 fields of view (FOV) selected at random. All videos and images were processed using Imaris, version 9.8 (Bitplane). Imaris software was used to quantify the accumulation of platelet aggregates or vWF deposits using surface tools and measuring the total area.

### In vivo inhibitor studies.

The platelet inhibitor ticagrelor (4 mg/kg) or vehicle (1× PBS) was delivered by oral gavage every 12 hours for 24 hours and immediately before infection. The ADAM10 inhibitor GI254023X (synthesized by Okeanos Technologies) (200 mg/kg/day) or vehicle (0.1 M carbonate buffer) was delivered i.p. every 12 hours for 3 days and immediately before infection. Six to 8 hours after infection, mice were imaged as above to assess platelet aggregation.

### Endothelial cell analysis.

HPAECs from Lonza were cultured in EGM-2 media (Lonza). Cells were pretreated with 20 μM ticagrelor (MilliporeSigma), 20 μM GI254023X (synthesized by Okeanos Technologies), or vehicle control (DMSO) for 16–18 hours prior to the experiment. A metalloprotease assay was performed by plating 2 × 10^4^ HPAECs in a 96-well plate 48 hours prior to experimentation. Cells were treated with 5 μg/mL Hla in unsupplemented media for 10 minutes, washed with metalloprotease buffer (50 mM Tris, 150 mM NaCl, 10 mM CaCl_2_), and incubated at 37°C with 10 μM ADAM10-specific fluorogenic peptide substrate II (BioZyme) for 30 minutes. Fluorescence intensity was read on a TECAN plate reader. For the cytotoxicity assay, HPAECs were exposed to 5 μg/mL Hla for 3 hours in unsupplemented media, and LDH release was measured using a cytotoxicity detection kit (Roche) according to the manufacturer’s protocol and read with a TECAN plate reader. For immunofluorescence, 2.5 × 10^4^ HPAECs were plated on IBIDI μ-Slide 8-well chamber slides and allowed to adhere for 72 hours. Cells were exposed to Hla (5 μg/mL) for 20 minutes and fixed in 4% paraformaldehyde. Cells were stained with an anti-vWF antibody (1:500, Dako, A0082) at room temperature for 1 hour or permeabilized with 0.1% Triton X-100 and stained with anti–VE-cadherin (1:100, Santa Cruz Biotechnology, F-8 SC-9989) at 4°C overnight. Following staining with an Alexa Fluor 594– or 488–conjugated secondary antibody, cells were visualized on a Zeiss Cell Observer inverted microscope with a color camera.

### Statistics.

Statistical analysis was performed using GraphPad Prism 8 (GraphPad Software). A log-rank (Mantel-Cox) test was used to compare survival curves, a 2-tailed, paired Student’s *t* test was used to compare 2 groups, a nested *t* test or nested 1-way ANOVA was used to compare platelet aggregates and vWF measurements between the control and treatment groups for in vivo imaging, and a 1-way ANOVA followed by Tukey’s multiple-comparison test was applied to determine the differences among 3 or more groups. The results were considered statistically significant at a *P* value of 0.05 or less.

### Study approval.

All animals in this study were housed according to Washington University in St. Louis IACUC guidelines, and all experimental procedures were approved by the IACUC of Washington University in St. Louis (protocol 19-1035).

### Data availability.

Values for all data points in graphs are reported in the Supplemental Supporting Data Values file.

## Author contributions

DNA and JBW designed and supervised the research. DNA performed the experiments and analyzed the data. DNA and MJM performed the 2-photon in vivo imaging. DNA and JBW wrote the manuscript.

## Supplementary Material

Supplemental data

Supplemental video 1

Supplemental video 2

Supporting data values

## Version 1 10/03/2023

In-Press Preview

## Version 2 12/01/2023

Electronic publication
