# Supplementary material for: Endothelial ADAM10 utilization defines a molecular pathway of vascular injury in mice with bacterial sepsis
Source: J Clin Invest. 2023 Dec 1;133(23):e168450. doi: 10.1172/JCI168450 (PMC10688991; doi:10.1172/JCI168450)
Supplement: Supplemental data [file jci-133-168450-s044.pdf]

Supplemental Data

Endothelial ADAM10 utilization defines a molecular pathway of vascular injury in in mice with bacterial sepsis

Danielle N Alfano, Mark J Miller, Juliane Bubeck Wardenburg

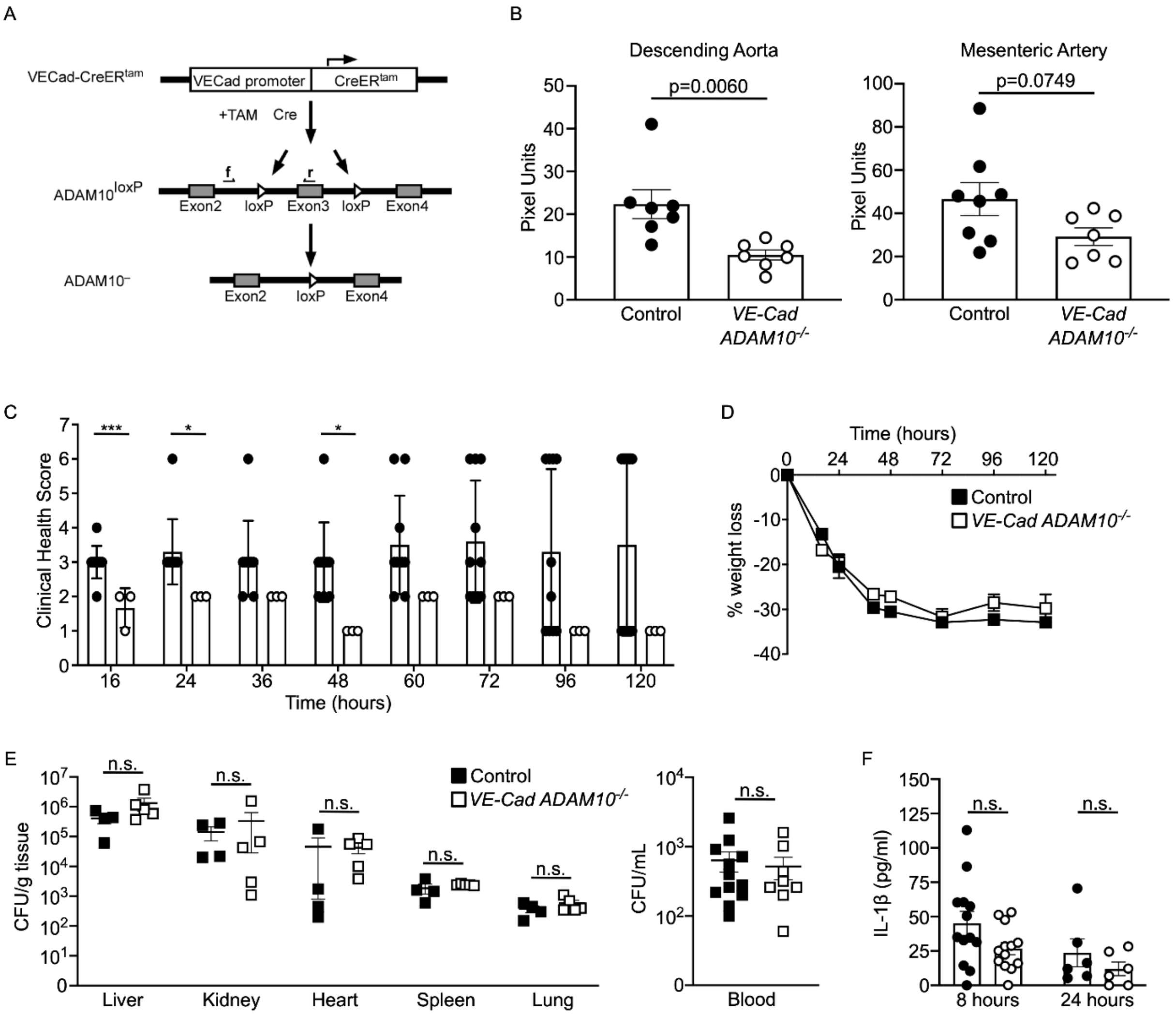

Supplemental Figure 1. Characterization of endothelial-specific ADAM10 knockout mice. (A) Genetic strategy to generate mice harboring a conditional deletion of *ADAM10* in endothelial cells using a vascular endothelial cadherin (VE-Cad) promoter-driven tamoxifen-inducible Cre-recombinase. **(B)** Quantification of mean red pixel intensity from control or *VE-CadADAM10*$^{-/-}$ mice to examine ADAM10 expression in descending aorta or mesenteric artery. Data represent 2-3 sections per vessel with 3 mice per group. Data represent mean ± SEM. **(C)** Representative clinical health scores from mice during lethal *S. aureus* sepsis. Data are represented as mean ± SD for each

time point. **(D)** Average calculated percent weight loss from baseline at designated timepoints post lethal *S. aureus* sepsis in control (n=8F) and *VE-Cad ADAM10*$^{-/-}$ mice (n=6F). Data representative of four independent experiments and represent mean ± SEM. **(E)** *S. aureus* colony-forming units (CFU) burden in organs and blood 24 hours after infection of control and *VE-Cad ADAM10*$^{-/-}$ mice. Data representative of two independent experiments in male and female mice. Data represent mean ± SEM. **(F)** Serum IL-1β analysis 8 and 24 hours post infection in female *VE-Cad ADAM10*$^{-/-}$ mice or controls. Data represent three independent pooled experiments. Data are represented as mean ± SEM. Statistical analyses are by unpaired 2-tailed t test for comparing two groups and 1-way ANOVA followed by Tukey's multiple comparisons among more than 3 groups, * $p \leq 0.05$, *** $p \leq 0.001$

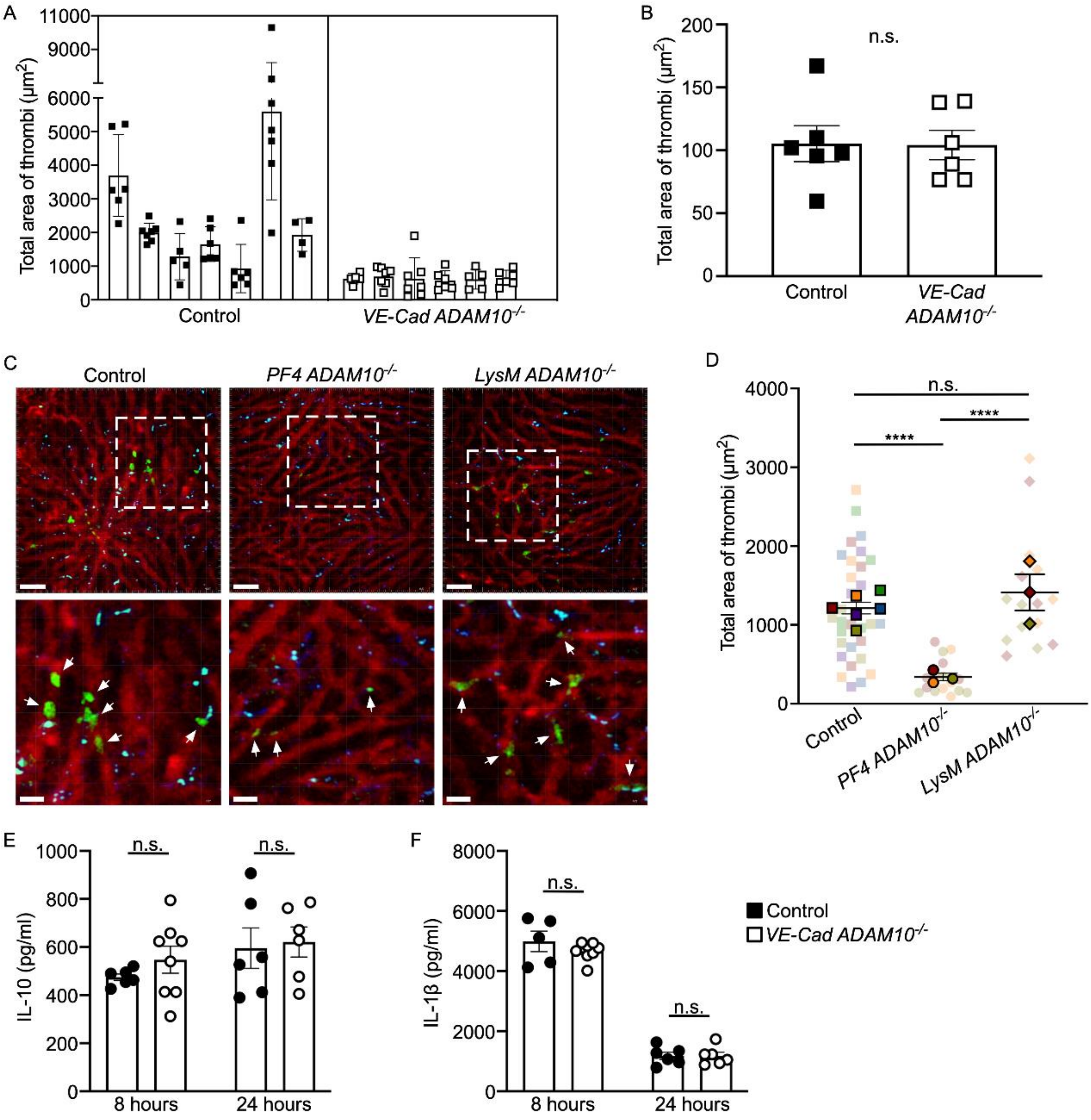

Supplemental Figure 2. Hla targets platelets and myeloid lineage cells in response to *S. aureus* to contribute to sepsis-associated injury. (A) Measurement of total area of platelet accumulation within the vasculature for each field of view within each mouse as infected in Figure 2, A and B. Data represented as mean ± SD. **(B)** Quantification of total area of platelet accumulation with the liver vasculature of uninfected control and *VE-Cad ADAM10*$^{-/-}$ female mice. Data represented as mean ± SEM. **(C)** Representative two-photon image of control, *PF4 ADAM10*$^{-/-}$, or *LysM*

ADAM10$^{-/-}$ mouse livers 6-8 hours after non-lethal *S. aureus* sepsis. Vasculature (red, Qdots655), platelets (green, GPIbβ). Scale bar of upper panels 50 µm. Lower panels display zoomed in image outlined by white dotted box with scale bar of 20 µm. White arrows denote thrombi. **(D)** Quantification of total area of platelet accumulation within the vasculature in mouse liver as treated in (C). Data represent 5-7 fields of view (FOV) per mouse in control (n=1M, 5F), *PF4 ADAM10*$^{-/-}$ (n=3F), or *LysM ADAM10*$^{-/-}$ (n=2M, 1F) mice per group ± SEM. **(E)** Liver IL-10 and **(F)** IL-1β analysis 8 and 24 hours post infection in female *VE-Cad ADAM10*$^{-/-}$ mice or controls. Data represent two independent pooled experiments. Data are represented as mean ± SEM. Statistical analyses by nested 1-way ANOVA for in vivo imaging among 3 groups and unpaired 2-tailed test for comparing 2 groups, ** $p \leq 0.01$, **** $p \leq 0.0001$

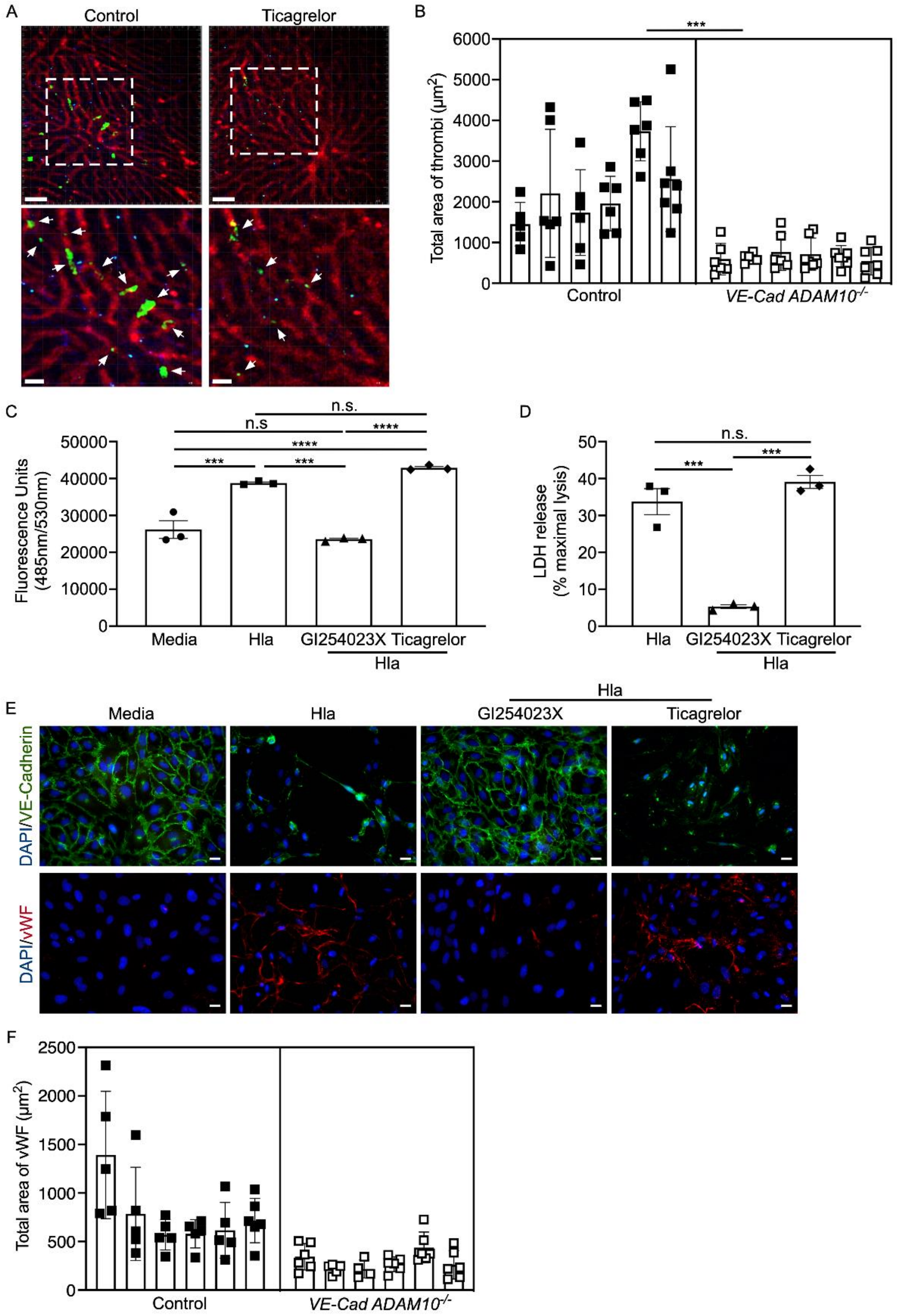

Supplemental Figure 3. P2Y12 inhibitor ticagrelor protects against *S. aureus* sepsis induced platelet aggregation but not Hla-mediated endothelial cytotoxicity.

(A) Representative two-photon image of livers from C57Bl/6 mice 6-8 hours after *S. aureus* sepsis pretreated with PBS vehicle or ticagrelor 24 hours before infection. Vasculature (red, Qdots655), platelets (green, GPIbβ). Scale bar of upper panels 50 µm. Lower panels display zoomed in image outlined by white dotted box with scale bar of 20 µm. White arrows denote thrombi. **(B)** Measurement of total area of platelet accumulation within the vasculature for each field of view within each female mouse as infected in (A). Data represented as mean ± SD. **(C)** Toxin-induced metalloprotease activity in HPAECs pre-treated with GI254023X, ticagrelor or vehicle stimulated with Hla in the presence of fluorogenic ADAM10 substrate for 30 min. **(D)** Hla sensitivity of HPAECs pretreated with GI254023X, ticagrelor or vehicle and quantified by LDH release assay where percent maximal lysis is calculated relative to detergent-lysed endothelial cells. Data in (C) and (D) are representative of the average of 3 technical replicates from 3 independent experiments represented as mean ± SEM. **(E)** VE-cadherin (green) and vWF (red) release from primary human pulmonary artery endothelial cells (HPAECs) following stimulation with Hla in the presence of vehicle control, ADAM10 inhibitor GI254023X or ticagrelor. DAPI (blue) denotes cell nuclei. Scale bar 20 µm. Images represent n≥2 experiments. **(F)** Measurement of total area of vWF accumulation within the vasculature for each field of view within each mouse as infected in Figure 2, F and G. Data represented as mean ± SD. Statistical analyses are by nested t-test for in vivo imaging and 1-way ANOVA when comparing 3 or more groups, ** $p \leq 0.01$, *** $p \leq 0.001$, **** $p \leq 0.0001$

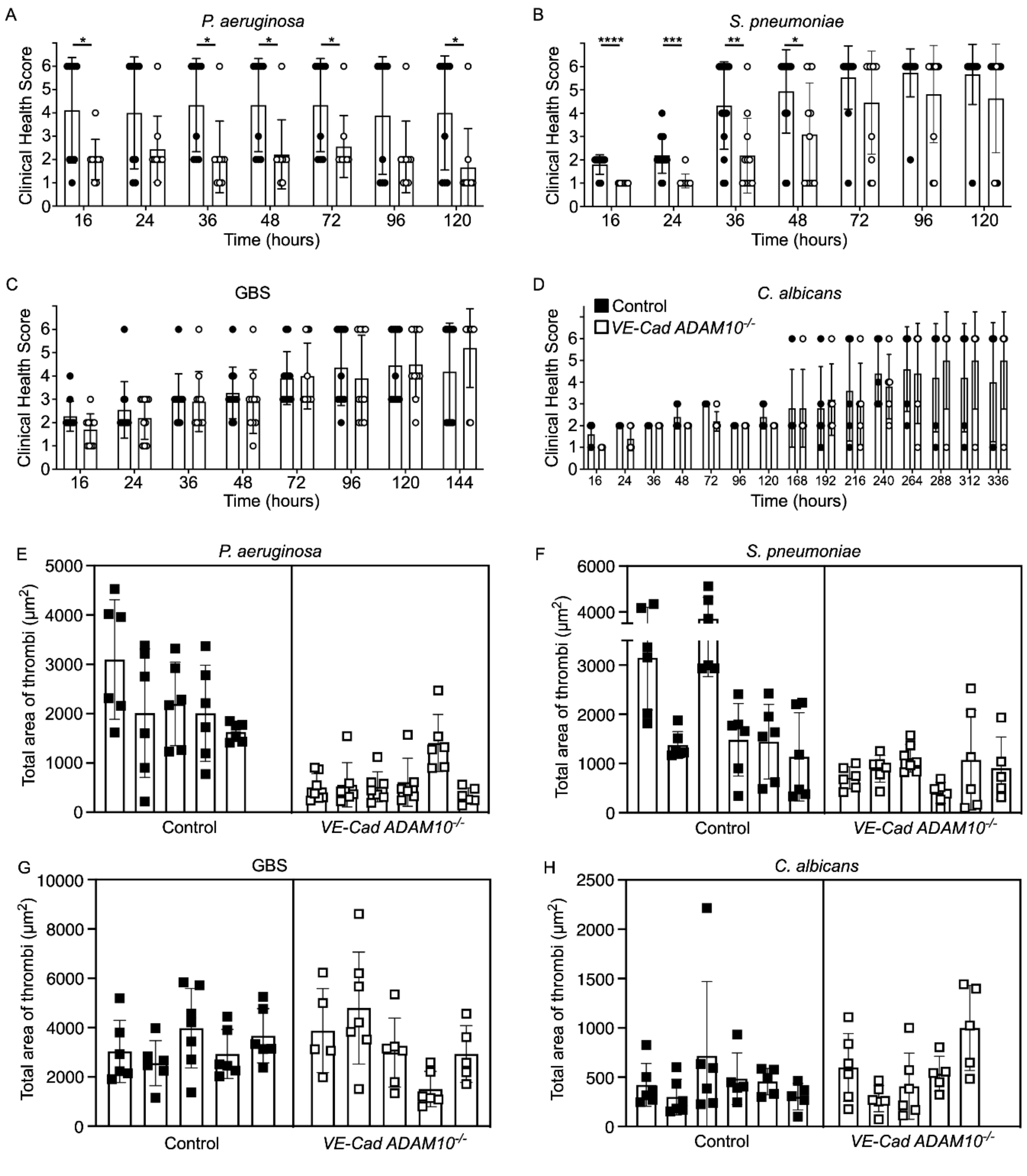

Supplemental Figure 4. Pathogen-specific lethal sepsis clinical scores and platelet aggregation. Representative clinical health scores from control and *VE-Cad ADAM10*$^{-/-}$ mice infected with lethal *P. aeruginosa* **(A)**, *S. pneumoniae* **(B)**, GBS **(C)**, or *C. albicans* **(D)**. **(E-H)** Measurement of total area of platelet accumulation within the vasculature for each field of view within each mouse as infected in Figure 4, A and B

with *P. aeruginosa* **(E)**, *S. pneumoniae* **(F)**, GBS **(G)**, or *C. albicans* **(H)**. Data represented as mean ± SD. Statistical analyses for clinical scores are by multiple t-tests, * $p \leq 0.05$, ** $p \leq 0.01$, *** $p \leq 0.001$, **** $p \leq 0.0001$

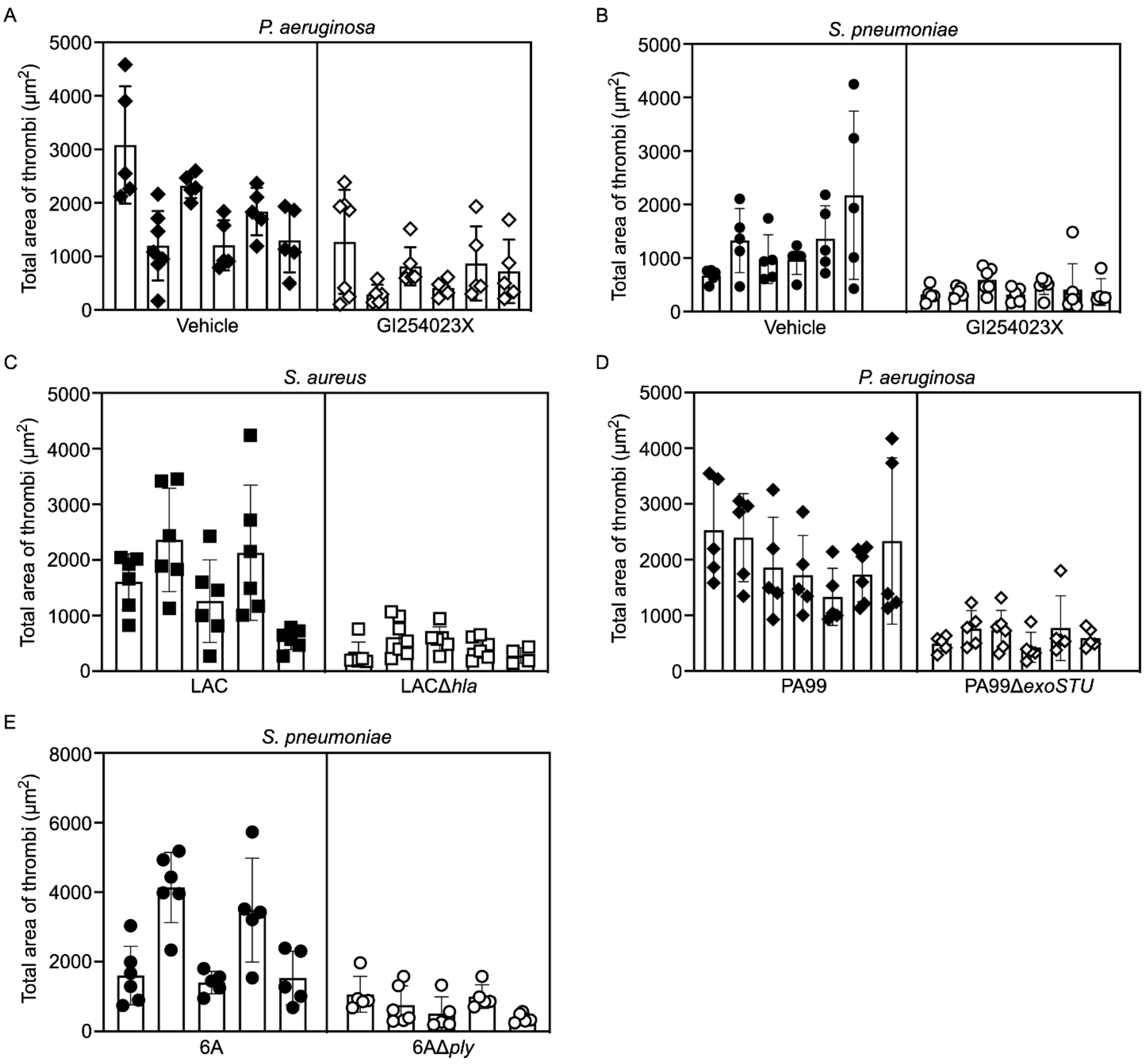

Supplemental Figure 5. Pathogen-specific lethal sepsis platelet aggregation with ADAM10 inhibitor or isogenic strains. (A & B) Measurement of total area of platelet accumulation within the vasculature for each FOV within each mouse as infected in Figure 5, A and B with *P. aeruginosa* **(A)** or *S. pneumoniae* **(B)**. **(C-E)** Measurement of total area of platelet accumulation within the vasculature for each FOV within each mouse as infected in Figure 6, A-D with WT and toxin null strains of *S. aureus* **(C)**, *P. aeruginosa* **(D)** or *S. pneumoniae* **(E)**. Data represented as mean ± SD.

Supplemental Table. Clinical health scoring system for in vivo sepsis model.

Score	Behavior
1	Animal is bright, alert, active, eating and drinking normally
2	Alert but somewhat less active, spontaneously moves about cage upon visual inspection
3	Animals huddled with ruffled fur, decreased or absent spontaneous movement upon visual inspection of cage; however will move when animal is touched; maintains righting reflex
4	Hunched and ruffled, animal does not move when touched, absent righting reflex
5	Recumbent
6	Death

Supplemental Video. Clinical behavior of mice during lethal sepsis. Video of clinical behavior of **(A)** wild-type and **(B)** *VE-Cad ADAM10*$^{-/-}$ mice 16 hours post infection with lethal *S. aureus* sepsis.
